# Role of SOST in Response to Mechanical Stimulation in Bone and Extraosseous Organs

**DOI:** 10.3390/biom15060856

**Published:** 2025-06-11

**Authors:** Minyou Chen, Wenjing Li, Le Lei, Lingli Zhang

**Affiliations:** 1 College of Athletic Performance, Shanghai University of Sport, Shanghai 200438, China; 2 School of Sport, Exercise and Health Sciences, Loughborough University, Loughborough LE11 3TU, UK

**Keywords:** SOST, extra-skeletal organs, osteoporosis, exercise, mechanical stimulation

## Abstract

Sclerostin (SOST) is a specific osteocyte protein. During the differentiation and proliferation of osteoblasts and osteoclasts, the high expression of SOST can inhibit bone formation and contribute to osteoporosis and the bone metastasis of malignant tumors. Most of the research on SOST has focused on bone cells, but studies have found that SOST is not a specific product of bone cells but that it is also expressed by articular chondrocytes. SOST can regulate the progression of osteoarthritis in bone and cartilage, promote subchondral bone sclerosis, and inhibit cartilage degeneration. A review of the literature found that SOST can not only regulate bone metabolism, but it is also expressed in cardiovascular, kidney, liver, and other tissues, influencing the occurrence and development of diseases in these organs and tissues. Studies have found that diseases of extra-bone organs, such as atherosclerosis, aneurysm, chronic kidney disease, and cirrhosis, may be related to the expression of SOST. Simultaneously, long-term exercise can reduce SOST levels, especially in areas of high bone strain. Prolonged exercise induces bone adaptation to mechanical stress, resulting in diminished responsiveness of bone cells to exercise and a reduction in serum SOST levels. Short-term acute exercise can elevate serum SOST levels, but these results are often limited by age, gender, and energy status. In general, serum SOST rises immediately after short-term acute exercise, returning to baseline or even decreasing after exercise.

## 1. Introduction

Sclerostin, a glycoprotein secreted by mature osteoblasts and encoded by the SOST gene, inhibits Wnt signaling and bone morphogenetic protein (BMP), negatively regulates bone formation, and plays an important role in skeletal development and the maintenance of bone mass. SOST was first thought to inhibit the BMP-dependent Smad pathway by interacting with BMP-competitive receptors; however, subsequent studies have found that SOST inhibits Smad1/5/8 signaling indirectly by inhibiting the Wnt signaling pathway, preventing excessive osteogenesis [1]. In addition, SOST expression is detected in various organs, including the bone, cartilage, kidney, liver, pancreas, heart, and placenta. Mechanical stress regulates the Wnt signaling pathway; in response to exercise (or mechanical stimulation), the expression of SOST is changed, exerting a regulatory effect on related diseases.

In this paper, we reviewed the literature, introduced the structure and function of SOST, summarized the molecular mechanism of SOST in extraosseous organs (such as the blood vessels, kidneys, and liver), and discussed the effect of exercise (or mechanical stimulation) on SOST. The results will provide a key target for future screening of gene-assisted therapy for diseases and offer new ideas for disease treatment and drug development after further investigation of SOST.

## 2. Structure and Function of SOST in Bone Homeostasis

SOST is mainly produced by osteoblasts, accounting for 90–95% of all bone-forming cells; upon secretion, SOST binds to the low-density lipoprotein receptor-related protein-4 (LRP4) on the osteoblast membrane, facilitating its retention within the bone cavity and serving as a negative regulator of bone formation [2]. Runt-related transcription factor 2 (Runx2), myocyte-specific enhancer factor 2C (Mef2c), and the osteoblast-specific transcription factor Osterix (Osx) positively regulate SOST expression [3], whereas the transcription factor NAD-dependent protein deacetylase sirtuin-1 (Sirt1) negatively regulates SOST expression [4]. In addition, SOST expression is regulated by epigenetic genes, such miRNAs, as well as DNA methylation [5].

**Figure 1:**
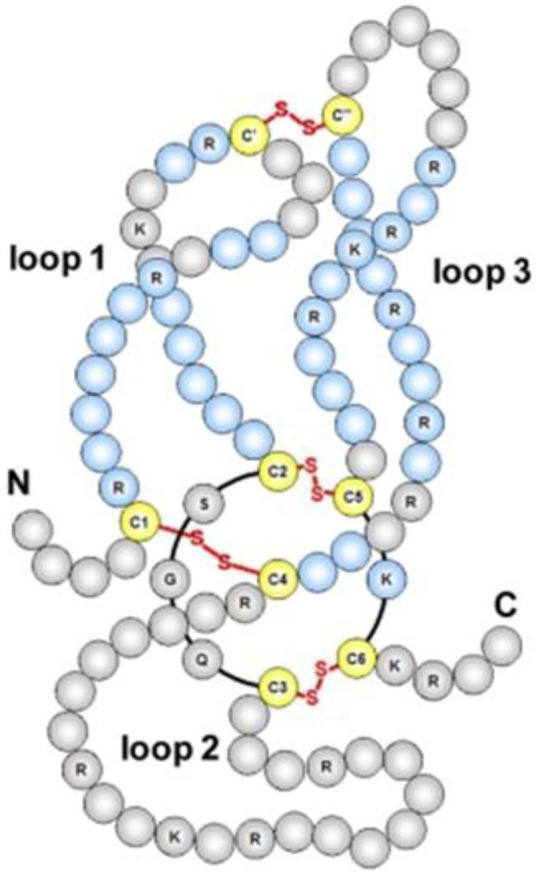
The “cystine knot” structure. The molecular structure of sclerostin shows the presence of disulfide bonds, resulting in a three-loop structure [10].

Mutations in the SOST gene produce a rare form of sclerosteosis (bone sclerosis), as well as Van Buchem disease, both characterized by increased bone mineral density (BMD) and excessive bone growth [6]. Sclerosteosis was first reported in 1958, whereas Van Buchem disease was identified in 1976. SOST is a secreted glycoprotein containing a “cystine knot” structure [7]. The SOST gene, localized in the D17S1787-D17S930 region of chromosome 17q12-21 in the human genome, encodes a protein homologous to the BMP-antagonistic DAN family. This protein contains 213 amino acids, possesses a molecular weight of 22 kD, and consists of a signal sequence for secretion and two putative glycosylation sites [8]. The “cystine knot” structure is a finger-like structure formed by two pairs of twisted, antiparallel β-strands, with the cystine knot located at the base. In the cystine knot motif, four highly conserved cysteine residues form two intrastrand disulfide bonds, forming a ring structure that typically consists of 8–14 residues. The conserved cysteine forms a third disulfide bond across the ring, producing an abnormally stable structural motif [9] (Figure 1).

SOST knockout mice exhibit elevated bone mass and demonstrate resistance to bone loss. When SOST binds to the surface receptor LRP5/6 on the osteoblasts, it negatively regulates the Wnt/β-catenin signaling pathway and inhibits bone formation in osteoblasts through competitive binding [11]. SOST also binds directly to LRP4, a membrane-bound protein that facilitates LRP5/6 interaction, which inhibits downstream Wnt signaling [12]. High expression of SOST inhibits bone formation, as evidenced by SOST’s ability to prevent the proliferation and differentiation of bone progenitor cells and osteoblasts, reduces the activation of mature osteoblasts, decreases bone mineralization, increases apoptosis of the osteoblasts, and maintains bone lining cells in a quiescent state, thereby regulating osteoblast maturation and osteoclast activity and stimulating bone resorption [11]. SOST can also regulate osteoblast differentiation and its bone formation by inhibiting the BMP and Wnt signaling pathways via its interaction with LRP5/6, which serves as a type 1 or type 2 receptor for BMP and a co-receptor for Wnt, respectively [13]. SOST not only inhibits osteoblast differentiation to reduce bone formation but also regulates the expression of RANKL/osteoprotegerin (OPG) [14]. However, SOST can promote extracellular matrix acidification and bone resorption [15]. The role of SOST in bone resorption is not well understood.

SOST is regulated by hormones (thyroxine, sex hormones, and glucocorticoids) and exercise (or mechanical stimulation), and parathyroid hormone (PTH) can inhibit SOST expression mediated by the cyclic adenosine monophosphate signaling pathway, resulting in a reduction in osteoblast numbers [16]. The imbalance of bone metabolism resulting from reduced estrogen has been linked to the upregulation of SOST expression [17]. A study on mice revealed that the application of mechanical stress inhibits SOST expression, but its removal results in the opposite effect [18].

## 3. Effects of SOST on Extraosseous Organs

SOST regulates bone metabolism, and it is also expressed in cardiovascular, renal, and hepatic tissues, influencing disease development in these organs and tissues. Atherosclerosis, aneurysm, chronic kidney disease (CKD), liver cirrhosis, and other extra-osseous organ diseases may be related to SOST expression. The following is a detailed description of the effects of SOST on extraosseous organs categorized by tissue and organ.

### 3.1. Effects of SOST on Blood Vessels

Atherosclerosis is a clinical manifestation of vascular aging, which mainly results from the abnormal proliferation of vascular smooth muscle [19]. The induction of vascular smooth muscle cells to calcify in vitro results in elevated levels of SOST [20]. SOST is present in atherosclerotic tissues, primarily functioning to inhibit the Wnt pathway by binding to the transmembrane Wnt coreceptors LRP-4, -5, and -6 [19]. Krishna et al. [21] found that SOST is expressed in the thoracic and abdominal aortas of mouse models of atherosclerosis, inhibiting angiotensin II (Ang II)-induced arterial calcification; they also observed its presence in the aorta of aneurysms and atherosclerosis. The upregulation of SOST effectively downregulates the expression of osteopontin (OPN), which facilitates inflammation and activates arterial calcification in Ang II mice, as well as OPG, which is involved in the activation of arterial calcification in Ang II mice. The upregulation of SOST effectively downregulates the expression of matrix metalloproteinase 9 (MMP9) and OPG, which are involved in the activation of arterial calcification in Ang II mice. The concentration of OPG is positively correlated with the development of hemangiomas and enhances the inflammatory response of vascular smooth muscle cells through conduitin S, MMP-2, and MMP-9. Additionally, SOST can inhibit the Ang II-induced formation of atherosclerosis and aneurysms, suggesting that SOST modulation may serve as a potential strategy for inhibiting these conditions [21]. Morales-Santana et al. [22] found that circulating SOST is elevated in patients with type 2 diabetes mellitus in the presence of atherosclerosis compared with patients in the non-atherosclerotic group. Moreover, serum SOST levels are positively correlated with aging in male patients with atherosclerosis, a correlation absent in females. Novo-Rodríguez et al. investigated the relationship between serum SOST levels and cardiovascular mortality, and they found that high SOST expression is associated with cardiovascular death. They also revealed that hyperglycemia, insulin resistance, and other cardiovascular risk factors cause endothelial damage, which in turn facilitate vascular calcification. SOST can be detected in the aorta of patients after surgery, even after aortic valve replacement, and SOST levels are upregulated in calcified vascular smooth muscle cells and calcified valve plaques [23,24]. Brandenburg et al. [25] suggested that the increase in SOST in the serum of patients under hemodialysis may result from heightened skeletal or extraosseous production of SOST, diminished renal clearance capacity, or a physiological adaptation of the vasculature to increased calcification. At present, the mechanism of action of SOST on vascular calcification and atherosclerosis needs to be further investigated, and the molecular mechanisms of SOST in atherosclerotic diseases and its specific role in the treatment of cardiovascular diseases require in-depth studies to provide new avenues for the treatment of these conditions.

### 3.2. Effects of SOST on the Kidney

Wnt signaling may play a potential role in CKD [26]. When renal function declines, SOST levels abnormally increase and inhibit the osteogenic Wnt/β-catenin signaling pathway, disrupting bone formation and bone resorption and triggering renal osteodystrophy in patients with CKD [27,28]. CKD–mineral bone disorder (CKD–MBD) is an abnormality of the mineral metabolism caused by CKD; it is characterized by renal dystrophy, vascular and soft-tissue calcification, an increase in the glomerular filtration rate (GFR), and a decline in bone mineral metabolism. As the GFR decreases, the concentration of SOST increases [27]. From the early stages of CKD, serum SOST in patients with CKD shows a progressive increase with decreasing renal function [29]. In patients with end-stage renal disease, circulating levels of SOST can reach 2–4 times those of the normal population [27]. Kidney transplantation is a viable option for patients with end-stage CKD. Zeng et al. [30] found that elevated serum SOST levels are an independent risk factor for death in kidney transplant recipients. Furthermore, SOST exhibits a U-shaped effect on vascular calcification and mortality. Although SOST is involved in the pathological changes associated with CKD–MBD, the source of its increase remains controversial. Peletier et al. [31] observed that from the third stage onwards, serum SOST is negatively correlated with renal function, inversely correlated with the estimated globular filtration rate (eGFR), and positively correlated with age. Reduced renal function and osteoporosis in the elderly may be linked to elevated SOST. Daniel Cejka et al. [32] found that the relative and absolute amounts of SOST excreted by the kidneys increase with decreasing eGFR. Moreover, SOST was detected in proximal tubular cells, suggesting that SOST is actively reabsorbed from urine. The increased renal excretion of SOST in patients with CKD may be due to increased SOST production and decreased tubular resorption. However, whether SOST inhibition would prevent bone loss or vascular calcification in CKD needs to be further investigated. Maré et al. [33] studied serum SOST levels in patients with end-stage renal disease (ESRD) and found that a serum SOST level assay can be used as a biomarker for bone metabolism disorders in patients with ESRD. Serum SOST and PTH in patients with ESRD have a negative correlation; PTH can act as a regulator of serum SOST, but the test results vary greatly with different testing equipment. Caution must be exercised when interpreting the data. SOST can provide a reference for the severity of renal inflammation and other diseases, and elevated serum SOST is a risk factor for renal disease, which may be the result of a combination of decreased renal excretory capacity, reduced tubular reabsorption, and local vascular secretion.

### 3.3. Effect of SOST on the Liver

Osteoporosis is a common complication in patients with chronic hepatitis, and about 75% of patients with chronic liver disease develop osteoporosis, which affects their quality of life and increases their risk of fracture [34]. Cirrhosis is an end-stage manifestation of chronic liver disease. Yumie et al. [35] found that patients with cirrhosis have higher levels of SOST than those of healthy individuals, and SOST levels are negatively correlated with serum albumin, which is a marker of hepatic dysfunction; moderate or severe hepatic dysfunction affects serum SOST levels. Alcohol abuse is an important predisposing factor for osteoporosis, and 35.9% of patients with alcoholic liver disease exhibit changes in bone metabolism and structure. Serum SOST levels are higher in patients with alcoholic cirrhosis than in patients with hepatitis B virus-associated cirrhosis, which may be due to age differences.

Wakolbinger et al. [36] found that SOST levels are low in patients with alcoholic liver disease, which may be due to the alcohol-induced apoptosis of bone cells. Zhou et al. [37] examined circulating SOST levels in patients with non-alcoholic fatty liver disease (NAFLD) versus healthy subjects, and they found that circulating SOST levels are significantly lower in patients with NAFLD than in control subjects; moreover, SOST levels in liver and bone are lower in HFD-fed mice than in control subjects. The circulating SOST levels in patients with NAFLD and healthy subjects are significantly lower than those in the control group, and the SOST levels in the liver and bone of HFD-fed mice are lower than those in the control group. Reduced SOST levels in patients with NAFLD primarily reflect the reduced secretion of SOST in bone tissues. Ehnert et al. [38] found that the main site of SOST increase in patients with primary biliary cirrhosis (BPC) is the bile ducts, and serum SOST levels are correlated with a reduction in BMD. SOST levels in BPC are correlated with decreased BMD, and the regulation of SOST levels via blood circulation affects bone metabolism, thereby contributing to the improvement of osteoporosis in patients with cirrhosis. SOST expression is correlated with the severity of inflammation and granulomas in the liver and gallbladder, and serum SOST levels in patients with early stage BPC show a decreasing trend over time. In advanced liver disease, bile acids gradually accumulate, and bilirubin decreases the mitochondrial activity of osteoblasts; serum SOST during the biliary nodule stage affects the proliferative capacity of osteoblasts [36]. Increased SOST levels in the bile and serum of macular patients reduces the survival and mineralization capacity of osteoblasts, and elevated SOST affects osteoblast formation during cholestasis [36]. Highly expressed SOST inhibits the Wnt signaling pathway during the differentiation and proliferation of skeletal cells, thereby inhibiting osteoblast proliferation and differentiation, as well as promoting programmed osteoblast death [11,38].

### 3.4. Effect of SOST on the Cartilage

**Figure 2:**
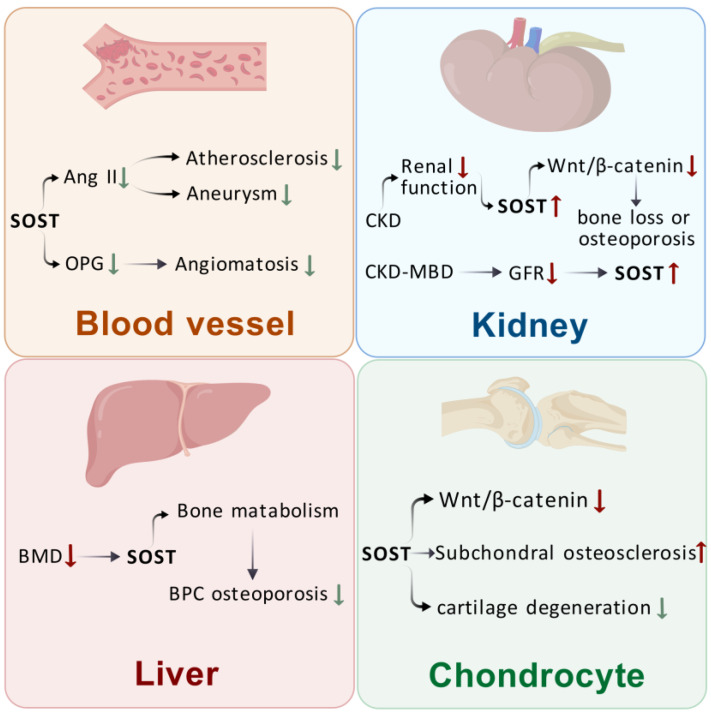
Effects of SOST on extraosseous organs. Sclerostin—SOST; angiotensin II—Ang II; osteoprotegerin—OPG; chronic kidney disease—CKD; glomerular filtration rate—GFR; bone mineral density—BMD; bone protection and control—BPC; chronic kidney disease–mineral bone disorder—CKD–MBD. (Created with BioGDP.com).

SOST is also expressed in mast cells of articular cartilage, and the regulation of its activity affects articular cartilage. Chen et al. [39] found that SOST, which is not specific to osteoblasts, is also expressed in articular chondrocytes and regulated by IL-1α. In osteoarthritis (OA), SOST is locally increased in chondrocytes. SOST not only inhibits Wnt/β-catenin signaling and the degradation of MMP, but it also diminishes the RNA levels of essential matrix components and enzyme inhibitors. SOST regulates OA in bone and cartilage, promotes subchondral osteosclerosis, and inhibits cartilage degeneration. Thompson et al. [40] found that the number and size of chondrocytes in animals decrease with age, alongside a reduction in the chondrocyte expression of SOST. DMM surgery on SOST transgenic mice resulted in accelerated OA progression in SOST overexpressing mice compared with that in WT control mice from the same litter [41]. This result was consistent with the findings obtained by Wafa Bouaziz et al. [42], who induced OA via DMM in SOST KO mice; they hypothesized that the regulation of Wnt/β-catenin signaling activity requires a certain level of SOST under pathological conditions. Roudier et al. [43] found that SOST expression remains unchanged in bone tissues of patients with knee OA. In SOST gene knockout mice, normally aged articular cartilage is not affected by changes in SOST levels. Thus, SOST may not be associated with knee OA, suggesting either a lack of association with cartilage destruction or the presence of other compensatory molecules in cartilage that mask the inhibitory effects of SOST (Figure 2).

In addition to the organs mentioned above, SOST is also associated with heart, lung, and brain disorders. One study found higher serum levels of SOST in myocardial infarction patients who developed cardiac remodeling after one year, suggesting that SOST may play a key role in the development of vascular remodeling [44]. Another study evaluated the correlation between SOST levels and the risk of cardiovascular disease in patients with type 2 diabetes mellitus; ultimately, they concluded that SOST may play a protective role against the development of atherosclerosis in patients with T2D by decreasing calcium deposition, decreasing the proliferative and inflammatory response, and promoting the cellular survival of the VSMC under calcified conditions [45]. Regarding the association of SOST with brain diseases, it has been suggested that plasma levels of the bone-derived protein SOST are positively correlated with cerebral Aβ load in a cognitively unimpaired elderly population [46]. In COPD, bronchial epithelial pathology activates the Wnt pathway, and SOST can block the Wnt signaling pathway; therefore, Amado [47] and others further investigated the link between SOST levels and COPD patients’ conditions, confirming that SOST levels correlate with body composition and lung function in COPD patients, and that lower levels of SOST predicted a higher risk of deterioration and hospitalization.

In summary, SOST sclerosing proteins are involved in the development and progression of a wide range of extraosseous organ diseases, including vascular, renal, hepatic, and chondrocytic diseases, by regulating multiple signaling pathways and physiological processes. SOST is expressed in several extraosseous organs, is associated with a variety of diseases, and may be able to serve as an observable indicator for a number of diseases, providing novel avenues for therapeutic intervention.

## 4. Exercise (or Mechanical Stimulation) Modulation of SOST

Literature has reported that physical activity enhances PBM in children and adolescents [48]. In particular, resistance training plays an important role in the development and preservation of bone tissue morphology, bone mass, and bone strength [49]. It also has a positive effect on BMD [50]. Increasing peak bone mass during the growth period in children and adolescents through exercise can prevent the risk of osteoporosis in middle-aged and older adults in later life [51]. Increased engagement in physical activity during adolescence promotes the accumulation of minerals in the skeletal structure, effectively prevents age-related bone loss, prevents osteoporosis in old age [49,52], reduces the risk of falls, and mitigates fractures. SOST is mainly secreted by osteocytes, which are the main cells responsible for mechanical signaling; the osteocytes will respond to external movement (or mechanical stimulation), subsequently altering SOST secretion [53]. Alterations in mechanical stimuli can diminish SOST expression in osteoblasts, facilitating bone formation and mineralization through the complex tensile and compressive stresses they generate. The application of mechanical loading stimuli can inhibit osteoblast differentiation and promote osteoclast formation upon the removal of the stimulus [54].

After reviewing the literature, Pickering et al. [55] studied the changes in serum SOST after acute exercise and found that young healthy women with no exercise habits exhibit elevated levels of systemic SOST after acute weight-bearing physical activity. This phenomenon may reflect a temporary response due to the longstanding perception that such physical activity will increase bone mass, with SOST acting as an osteoclast-secreted inhibitor of bone formation. Exercise-induced stimulation prompts the blood to release previously synthesized SOST, potentially linked to physiological regulation by the kidneys, which increases the tubular reabsorption of SOST by decreasing its excretion. Jürimäe, J. et al. [56] examined male athletes engaged in prolonged rowing and found an increase in serum SOST concentration after endurance training; exercise-induced changes in SOST may serve as a basis for monitoring exercise metabolism. Kouvelioti et al. [57] studied high-intensity interval exercise in women and found that the increase in serum SOST after exercise may result from the release of anchored SOST from osteoblasts into the bloodstream, rather than from a short-term increase in SOST gene expression. They then compared serum SOST expression in men and women after high-intensity interval exercise and observed that SOST increases in both sexes at 5 min post-exercise, with a greater increase in men than in women; thus, SOST is significantly correlated with the corresponding changes in inflammatory cytokines [58]. Both experiments led to similar conclusions, i.e., exercise caused an increase in blood flow to the skeleton and an increase in SOST at 5 min post-exercise, but SOST concentrations at 1, 24, and 48 h returned to pre-exercise levels, suggesting that the immediate rise in SOST post-exercise may lack metabolic significance.

Exercise affects SOST differently in males of varying ages. Falk et al. [59] compared changes in SOST after a single session of high-intensity exercise in boys and young adult males during late childhood and early adolescence. Their findings showed that young adult males experience an increase in SOST levels immediately after exercise, whereas boys do not exhibit any change. This discrepancy was hypothesized to result from the critical stage of physical development in boys, which is essential for bone mass accrual. An increase in SOST would enhance bone resorption, whereas the absence of such an increase would confer a protective effect on the boys, moderating the cellular response to exercise. Furthermore, an increase in bone-specific alkaline phosphatase, which promotes bone formation, was observed 24 h after exercise.

Prolonged exercise reduces SOST levels, especially in areas subjected to high skeletal strain. Exercise was found to reduce bone resorption and SOST-mediated inhibition of bone formation after 9 weeks of running–bench interval training and free-fall exercise in rats [53]. Liao et al. [27] investigated the effect of 8 weeks of moderate-intensity exercise on serum SOST levels in rats with CKD and found that exercise training mitigates the rise in serum SOST; moreover, they observed no change in the rate of bone formation but reported an improvement in bone resorption after the intervention. Exercise improves BMD and bone microarchitecture in mild CKD rats by inhibiting SOST but does not alter the serum mineral content. Hinton et al. [60] studied the effect of 12 months of resistance or jump training on serum SOST levels in men, revealing a significant reduction of 7% from baseline to the conclusion of the intervention. Their findings indicated that exercise interventions diminish circulating SOST as BMD increases over time. This result was consistent with osteoblastic SOST expression and demonstrated that exercise (mechanical stimulation) increases bone mass. Conversely, prolonged reduction in mechanical stimulation increases serum SOST levels. Spatz et al. [61] studied changes in serum SOST in healthy adult males after 90 days of bed rest and found an increase of 20% in SOST and a decrease of 2–4% in lower extremity BMD within 2–3 weeks of bed rest.

**Figure 3:**
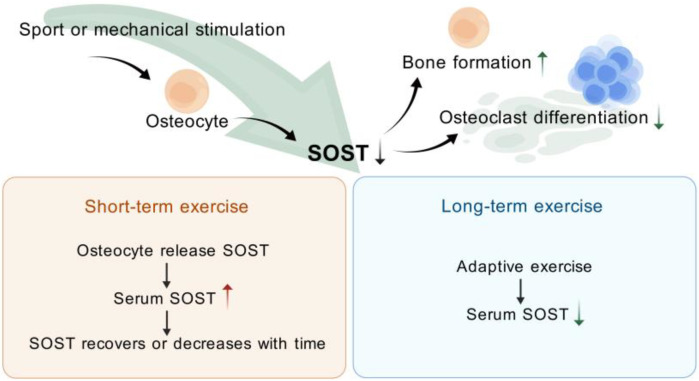
Exercise (or mechanical stimulation) modulation of SOST. (Created with BioGDP.com).

Short-term acute exercise stimulates an increase in serum SOST, but these findings are often limited by age, gender, previous training history, and energy status. Participation in these exercises may result in changes in body weight, calcium ion status, and energy balance, which can affect SOST release. In general, serum SOST levels increase immediately after short-term acute exercise and return to baseline or may decline even further. Prolonged exercise induces skeletal adaptation to mechanical stress, diminishes osteoblast response to exercise, and lowers serum SOST levels. Conversely, a prolonged reduction in mechanical stress elevates serum SOST, which was also consistent with the regulation of bone metabolism via the Wnt signaling pathway (Figure 3).

Under the influence of exercise or mechanical stimulation, osteoblasts cause changes in SOST secretion. After short-term exercise, serum SOST levels increase. After prolonged adaptation to exercise, serum SOST expression levels decrease.

## 5. Conclusions

SOST affects bone formation via the Wnt/β-catenin signaling pathway and bone resorption via RANKL/OPG regulation. Given that it is expressed in multiple extraosseous organs, it is also found to be associated with multiple extraosseous organ diseases. Mechanical stress regulates the expression of SOST; short-term acute exercise increases serum SOST levels, whereas prolonged exercise decreases serum SOST levels. As an important regulator of skeletal endocrinology, SOST is expressed in various organs and significantly influences glucose and lipid metabolism. This feature is the focus of future research on skeletal endocrinology and offers novel insights for the treatment of multiple organ sclerosis.

## Data Availability

Not applicable.
